# Role of Bruton's Tyrosine Kinase in B Cell Development

**DOI:** 10.1155/2001/28962

**Published:** 2001

**Authors:** Alex Maas, Rudolf W. Hendriks

**Affiliations:** 1 Department of Cell Biology and Genetics Erasmus University Rotterdam PO Box 1738 Rotterdam 3000 DR The Netherlands; 2 Department of Immunology Erasmus University Rotterdam PO Box 1738 Rotterdam 3000 DR The Netherlands

**Keywords:** B cell development, B cell receptor, Btk, immunodeficiency, XLA, *xid*

## Abstract

X-linked agammaglobulinemia (XLA) is one of the most frequent inherited immunodeficiency diseases in man and is characterized by an almost complete arrest of B cell differentiation at the pre-B cell stage. The gene defective in XLA encodes the cytoplasmic signaling molecule Bruton's tyrosine kinase (Btk). Next to the CBA/N strain of mice, carrying a single amino acid substitution mutation in the Btk gene, which results in the X-linked immunodeficiency (*xid*) phenotype, additional mouse models have been developed to study the role of Btk *in* *vivo*. This review discusses the analyses of Btk null-mutants, obtained by gene targeting in embryonic stem cells, and transgenic mice that express wild-type or mutated forms of the Btk gene. These studies provided information on the function of Btk at several important checkpoints throughout B cell development. Analyses of the mouse models indicated that Btk is not essential for pre-B cell receptor signaling in the mouse. By contrast, Btk-mediated B cell receptor signaling appears to be required for the survival of immature B cells in the bone marrow, that have performed a successful immunoglobulin (Ig) L chain locus rearrangement, resultirig in the expression of a non-autoreactive Ig on the membrane. Btk is also shown to be involved in signaling pathways that govern the development of peripheral B cells, including follicular entry, follicular maturation and plasma cell differentiation.


*Developmental Immunology*, 2001, Vol. 8(3-4), pp. 171-181
Reprints available directly from the publisher
Photocopying permitted by license only

© 2001 OPA (Overseas Publishers Association)
N.V. Published by license under
the Harwood Academic Publishers imprint,
part of The Gordon and Breach Publishing Group,
a member of the Taylor and Francis Group

### Role of Bruton's Tyrosine Kinase in B Cell Development

ALEX MAAS[a] and RUDOLF W. HENDRIKS[ab*]

[a]*Department of Cell Biology and Genetics and* [b]*Department of Immunology, Erasmus University Rotterdam, PO Box 1738, 3000 DR, Rotterdam, The Netherlands*

X-linked agammaglobulinemia (XLA) is one of the most frequent inherited immunodeficiency diseases in man and is characterized by an almost complete arrest of B cell differentiation at the pre-B cell stage. The gene defective in XLA encodes the cytoplasmic signaling molecule Bruton's tyrosine kinase (Btk). Next to the CBA/N strain of mice, carrying a single amino acid substitution mutation in the Btk gene, which results in the X-linked immunodeficiency (*xid*) phenotype, additional mouse models have been developed to study the role of Btk *in vivo*. This review discusses the analyses of Btk null-mutants, obtained by gene targeting in embryonic stem cells, and transgenic mice that express wild-type or mutated forms of the Btk gene. These studies provided information on the function of Btk at several important checkpoints throughout B cell development. Analyses of the mouse models indicated that Btk is not essential for pre-B cell receptor signaling in the mouse. By contrast, Btk-mediated B cell receptor signaling appears to be required for the survival of immature B cells in the bone marrow, that have performed a successful immunoglobulin (Ig) L chain locus rearrangement, resulting in the expression of a non-autoreactive Ig on the membrane. Btk is also shown to be involved in signaling pathways that govern the development of peripheral B cells, including follicular entry, follicular maturation and plasma cell differentiation.

*Keywords:* B cell development, B cell receptor, Btk , immunodeficiency, XLA, *xid*

### X-LINKED AGAMMAGLOBULINEMIA AND X-LINKED IMMUNODEFICIENCY

B cell development is regulated by multiple signals derived from stromal cell contact, cytokines, antigens and helper T cells. Biochemical analyses and *in vivo* gene targeting experiments have implicated tyrosine kinases as key regulators of many of these signaling pathways (Satterthwaite and Witte, 1996). Bruton's tyrosine kinase (Btk) is one of the non-receptor protein tyrosine kinases involved in regulating the B cell development and function (Tsukada et al., 1993, Vetrie et al., 1993; Rawlings et al., 1993, Thomas et al., 1993; for review see: Sideras and Smith, 1995, Desiderio, 1997, Conley and Cooper, 1998; Mohamed et al. 1999). Btk is a 659 amino acid protein and belongs to a subfamily of tyrosine kinases, which also includes Itk, Tec and Bmx. Members of this family are expressed in haematopoietic cells and are all involved in signal transduction pathways activated by growth or differentiation factors. Btk contains, in addition to the *Src* homology domains SH2 and SH3 and a single C-terminal catalytic domain, a unique pleckstrin homology (PH) domain at the N-terminus and an adjacent proline- and cysteine-rich Tec homology domain. The biochemistry of Btk activation after B cell receptor signaling has recently been reviewed (Mohamed et al., 1999).

* Corresponding Author.

Defects in the Btk protein result in the B cell differentiation defects X-linked agammaglobulinemia (XLA; Bruton's disease) in man and X-linked immunodeficiency (*xid*) in mice. XLA patients manifest recurrent bacterial infections due to a profound reduction of serum immunoglobulin (Ig) of all classes. They have very low B cell numbers in the peripheral blood, and those few B cells present exhibit an immature $IgM^{high}$ phenotype (Conley 1985; Campana et al., 1990). If stimulated with anti-CD40 *in vitro* these B cells are able to proliferate and differentiate into specific Ig-producing cells (Nonoyama et al., 1998). Plasma cells are almost completely lacking. Because the numbers of pre-B cells in the bone marrow are not significantly reduced, XLA reflects impaired developmental progression or increased cell death at the transition from pre-B to immature B cells in the bone marrow. Since the discovery of the *Btk* gene, a large variety of mutations, including single nucleotide substitutions, insertions and deletions, distributed over the entire *Btk* coding region have been characterized in 471 unrelated XLA families (Vihinen et al., 1999). There is phenotypic heterogeneity among patients, even among patients from single XLA pedigrees (Bykowsky et al., 1996, Holsinki-Feder et al., 1998, Vihinen et al., 1999). The arrest of B cell development in XLA patients is not precisely defined and was shown to vary between patients (Campana et al., 1990). So far, it has not been possible to correlate severity of the phenotypic presentation with the genotype. Recently developed techniques, such as single-cell PCR analysis, and a detailed characterization of cell surface markers in XLA bone marrow samples could help to investigate the role of Btk in human B cell development. However, the large genetic variety, differences in habitat or therapeutic interventions between patients might complicate these studies. Therefore, mouse models could serve as an alternative to study the role of Btk and the effect of *Btk* mutations in B cell development, since they exhibit a uniformly genetic background and can be kept under comparable conditions.

Since the early 1970s, an impressive amount of data has been accumulated concerning the functional defects in the CBA/N strain of mice, carrying the X-linked immunodeficiency (*xid*) mutation (Wicker and Scher, 1986). Shortly after the identification of the Btk gene, it was shown that these mice have a mutation in the *Btk* PH domain, of the highly conserved $Arg_{28}$ residue into cysteine (Rawlings et al., 1993, Thomas et al., 1993). The *xid* phenotype in the mouse is less severe than XLA in humans (Table I). These mice have ~50% fewer B cells in the periphery and the residual cells exhibit an unusual $IgM^{high}$ $IgD^{low}$ phenotype. They lack the $CD5^{+}$ B-1 B cell population and the IgM and IgG3 serum levels are severely reduced. Btk-deficient B cells do not enter S phase after anti-IgM stimulation *in vitro*. Although the immune response of *xid* mice to T cell-dependent antigens is undisturbed, they fail to make antibodies to T cell-independent type 2 antigens *in vivo*.

The milder phenotype of murine *xid*, when compared with human XLA, cannot be explained by the nature of the mutations involved. Mutation of the same $Arg_{28}$ amino acid has been observed in patients with classic severe XLA phenotypes (de Weers et al., 1994a; Vihinen et al. 1999). Furthermore, the analysis of mice deficient for Btk in their germline, which were generated by gene targeting, showed that the complete absence of Btk protein also results in the mild *xid* phenotype (Khan et al., 1995; Hendriks et al., 1996). The molecular basis of the differences in phenotype between the two species is not well understood, although it is shown that the severity of the *xid* mutation is dependent on the genetic background of mice (Bona et al., 1980, Khan et al., 1996). Differences can partially be caused by the relative contributions of alternative pathways of B cell differentiation in a Btk-independent manner. An alternative explanation would be that there are differences in availability or functionality of Btk-relatives that may compensate for the absence of functional Btk, as expression of wild-type Btk or other Tec-family members can restore $Ca^{2+}$ fluxes in cell lines derived from XLA patients (Fluckinger et al., 1998). In this context, the B cell receptor (BCR)-induced hydrolysis of phospholipids in XLA cell lines was found to be severely reduced, leading to a strong reduction in $Ca^{2+}$ flux, whereas *xid* B cells demonstrate only a two-fold reduction in phosphatidyl-inositide hydrolysis (Fluckinger et al., 1998, Takata & Kurosaki, 1996).

TABLE I Comparison of the phenotypes of human XLA and murine *xid*

| | |
|---|---|
| *Differences between XLA and xid*[a] : | |
| 1. | The pre-B to immature B cell transition: almost complete arrest in XLA, and only a mild selective disadvantage for Btk-deficient cells in *xid*. |
| 2. | Peripheral B lymphocyte population: absent or very low in XLA, and only a limited reduction (~50%) in *xid*. |
| 3. | Ig levels in the serum: very low levels of all isotypes in XLA, reduced levels of IgM and IgG3 and generally normal levels of other isotypes in *xid*. |
| 4. | *In vivo* responses to T cell dependent antigens: low but detectable in XLA, and normal in *xid*. |
| *Similarities of XLA and xid*: | |
| 1. | Intrinsic B cell defect; other haematopoietic lineages are unaffected. |
| 2. | Early B cell development up to the pre-B cell stage in the bone marrow is normal. |
| 3. | Residual peripheral B cells have an immature $IgM^{high}$ surface phenotype. |
| 4. | T cell independent antibody responses *in vivo* are lacking. |
| 5. | Normal responses to anti-CD40 stimulation *in vitro*. |
| 6. | Heterozygous female carriers are normal, but have non-random X chromosome inactivation in the mature B cell population. |
| 7. | Phenotypic heterogeneity between patients within single XLA families and between families; expression of the *xid* phenotype is dependent on the background of the mouse strain. |

a. See text for details (Conley, 1985; Wicker and Scher, 1986; Sideras and Smith 1995; Kahn et al. 1995; Hendriks et al., 1996; Nonoyama et al., 1998).

In spite of the obvious differences in severity of the phenotype, Btk appears to be a conserved key factor involved in both murine and human B cell development. Btk has been shown to be involved in the BCR signal transduction pathways in both human (De Weers et al., 1994b, Hinshelwood et al., 1995) and murine (Aoki et al., 1994, Saouf et al., 1994) B cells. The genomic organization of the murine *Btk* gene is very homologous to the human *Btk* gene and the Btk proteins share 99.3% homology (Tsukada et al., 1993). Transgenic human *Btk* could fully compensate for the absence of murine Btk (Drabek et al., 1997, Dingjan et al., 1998, Maas et al., 1997, 1999), indicating that the essential sites for Btk interaction with other signal transduction components are conserved between human and mouse. In addition, large scale comparative sequence analysis of the human and murine *Btk* loci revealed clusters of sequence conservation in non-coding regions throughout the loci (Oeltjen et al., 1997), which may play an essential role in the complex gene regulation. When human Btk was expressed under the control of the endogenous *cis*-acting elements in a 340 kb or a 240 kb transgene construct, the expression pattern of transgenic Btk paralleled that of the endogenous Btk (Maas et al., 1997, A.M., unpublished results). We conclude that also *cis*-acting elements that regulate Btk expression have been conserved. Finally, *in vivo* competition experiments between B cells either expressing the wild-type *Btk*$^{+}$ gene or a targeted disrupted *Btk*$^{-}$ allele (see below), demonstrated that Btk$^{-}$ murine pre-B cells are also hampered in their progression to the immature B cell stage (Hendriks et al., 1996). Thus, XLA and xid may well involve the same stages of B cell development, but with quantitative differences between the species (Table I).

### TARGETED MUTATION OF BTK BY INSERTION OF A LACZ REPORTER

To determine the stage in B cell development at which defects in Btk become apparent, a mouse model was generated in which the *Btk* gene was inactivated through a targeted in-frame insertion of a β-galactosidase (*lacZ*) reporter (Hendriks et al., 1996). The *xid* phenotype in these mice confirmed the earlier findings in Btk null-mutant mice (Khan et al., 1995; Kerner et al., 1995) that the elimination of Btk function does not lead to an almost complete block in B cell development, which is typical for XLA in man.

The presence of the *lacZ* reporter enabled us to determine the Btk expression profile *in vivo*. We found that Btk is expressed throughout B cell development, from the pro-B cell stage to the most mature $IgM^{low}IgD^{high}$ peripheral B cell and activated B lymphoblasts stage. Btk expression is down-regulated in plasma cells, and was also not found in T cells or NK cells. These patterns were consistent with other Btk expression pattern studies, either in cell lines and leukemias (De Weers et al., 1993, Genevier et al., 1994, Smith et al., 1994, Tsukada et al., 1993) or *in vivo* using intracellular flow cytometric Btk detection (Maas et al., 1999). Expression of the Btk gene was not restricted to the B cell lineage. In the bone marrow, the $ER\text{-}MP20^{high}$ precursor cells of the monocyte lineage showed high *lacZ* activity, whereas the $ER\text{-}MP20^{medium}$ fraction of granulocyte precursors manifested heterogeneous levels of *lacZ* expression (Figure 1). In the erythroid lineage ($Ter\text{-}119^{+}$; $ER\text{-}MP20^{low}$), *lacZ* activity was mainly detected in the most immature population of large erythroid precursors (Figure 1). This wide expression pattern of Btk suggests a role for Btk in multiple signaling pathways. Indeed, apart from its role in BCR-signaling, Btk has been implicated as a mediator of signals from the interleukin 5 receptor (IL-5R), IL-6R and CD38 in B lymphocytes, the FcεRI in myeloid cells, as well as the collagen receptor glycoprotein VI in platelets (Sideras and Smith, 1995, Wahl et al., 1997; Quek et al., 1998). It was recently reported that Btk-deficient macrophages produce less nitric oxide than wild-type macrophages in response to a variety of stimuli (Mukhopadhyay et al., 1999). Despite these findings, Btk is only essential in B cell development and cells from other haematopoietic lineages do not appear to be affected in XLA nor in *xid* (Sideras and Smith, 1995).

Using the *Btk⁻/lacZ* mouse model it was also possible to detect selective disabilities of Btk-deficient cells in each successive step of B cell development in an *in vivo* competition strategy (Hendriks et al., 1996). Due to the phenomenon of random x chromosome inactivation, B-cell precursors in $Btk^{+/-}$ heterozygous female mice express either the wild-type $Btk^{+}$ allele or the targeted $Btk^{-}$*/lacZ* allele. The first selective disadvantage of Btk-deficient cells became apparent at the transition from small pre-B into immature B cells in the bone marrow (Hendriks et al., 1996). Moreover, the observed accumulation of $Btk^{-}$ cells within a small subpopulation of $CD43^{-}$ pre-B cells (R.W.H., unpublished observation) further indicated that $Btk^{-}$*/lacZ* cells were defective in the pre-B to B cell transition. A second maturation arrest was found during the maturation from $IgM^{high}IgD^{low}$ to $IgM^{low}IgD^{high}$ stages in the periphery. These findings also implied that Btk is not involved in early signaling pathways essential for the proliferation or differentiation of pro-B or early pre-B cells, which is stroma cell-dependent and driven by cytokines such as IL-7. The fraction of *lacZ*-expressing cells in $Btk^{+/-}$ heterozygote female mice did not change during the migration of the immature B cells from the bone marrow to the spleen, indicating that Btk expression is not relevant for this migration process (Hendriks et al., 1996).

### CORRECTION OF THE XID PHENOTYPE BY TRANSGENIC EXPRESSION OF BTK

We and others have generated transgenic mice in which expression of Btk is driven by various promoters, in order to correct the *xid* phenotype in Btk-deficient mice. In these experiments, also the minimal dosage required for Btk function was studied and the effects of Btk overexpression on B cell development and B cell function were analyzed.

Transgenic expression of the human Btk gene was driven by the murine MHC class II Ea gene locus control region (LCR), which was shown to provide position-independent and copy-number dependent expression from the pre-B cell stage onwards (Drabek et al., 1997). When these transgenic mice were mated onto a Btk-deficient background, correction of the *xid* B cell defects was observed. B cell differentiated to $IgM^{low}IgD^{high}$ stages in spleen or lymph nodes and peritoneal $CD5^{+}$ B-1 B cells were present. In the serum the levels of IgM and IgG3 were in the normal ranges, and B cell responses to the T cell independent type II antigen di-nitrophenol-Ficoll were present. A

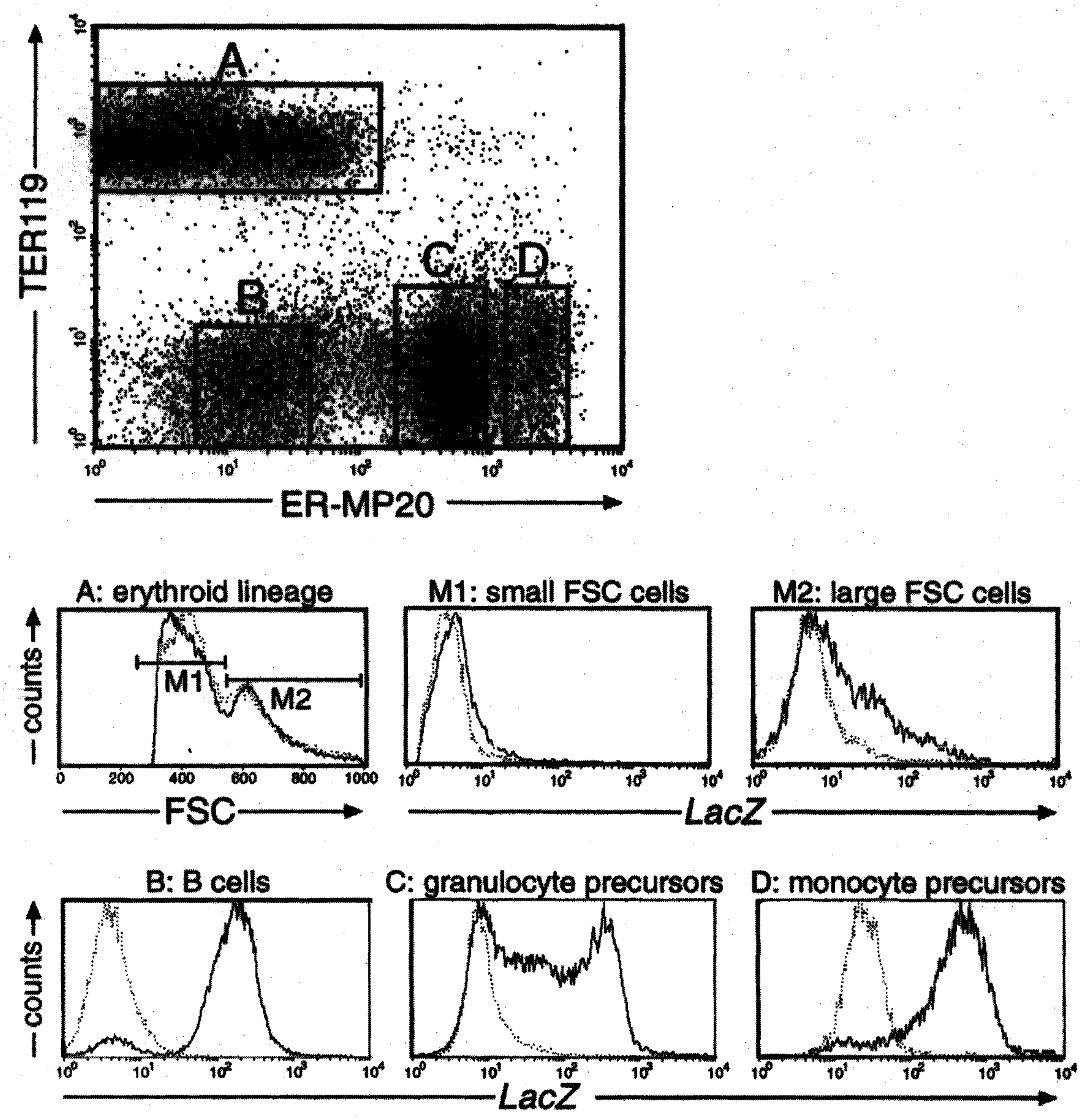

FIGURE 1 The expression of Btk in various bone marrow populations in the mouse. Surface profile of the ER-MP20 and TER-119 markers (top). The gated populations were analyzed for lacZ expression and the results are displayed as histograms. The Ter-119$^{+}$ erythroid cells were subdivided on the basis of forward scatter (FSC) profiles into small and large cells. Solid lines represent cell populations of Btk$^{-}$/lacZ mice; dashed lines represent the background lacZ activity as determined in wild-type mice comparable rescue was also observed in heterozygous Btk$^{+/-}$ female mice in those B cells that were Btk-deficient as a result of the inactivation of the X chromosome carrying the intact endogenous Btk gene. This apparent correction of the Btk-deficient phenotype by expression of Btk from the pre-B cell stage onwards indicates that Btk is not essential in the very early stages of B cell development.

Using the Ig H chain enhancer and promoter to rescue the *xid* phenotype, it was shown that murine Btk expression that equaled ~25% of endogenous levels was sufficient to restore normal numbers of B cells in the spleen, which were phenotypically mature (Satterthwaite et al., 1997). However, serum Ig levels, T cell independent type II responses, $CD5^+$B-1 B cell development and *in vitro* responses to anti-IgM stimulation remained significantly impaired in these animals. These data indicated that the development of mature conventional B cells, the development of $CD5^+$ B-1 B cells, and B cell responses to antigen *in vivo* may require higher levels of Btk activity.

In a next series of experiments, we increased the expression levels of transgenic Btk by including genomic DNA from the Btk gene (16 out of 18 introns) and the endogenous 3′ untranslated region (Dingjan et al. 1998). Although we observed a significant overexpression (up to ~14x in the spleen of one of the transgenic lines generated), this did not appear to result in adverse effects on B cell development or function. Complete correction of all *xid* features was also observed by transgenic expression of the wild-type human Btk under the control of the B-cell specific CD19 promoter region or endogenous regulatory sequences present on a 340 kb yeast artificial chromosome Btk construct (Maas et al., 1997, 1999). Therefore it is concluded that Btk overexpression per se does not lead to significant activation of downstream signaling pathways in the mouse.

### EXPRESSION OF THE E41K BTK MUTANT IN TRANSGENIC MICE

It has been shown that Btk tyrosine phosphorylation and the *in vitro* kinase activity of Btk increase upon BCR stimulation (Saouf et al., 1994; De Weers et al., 1994; Aoki et al., 1994), placing Btk in the BCR signal transduction pathway. BCR engagement leads to activation of phosphatidyl-inositol-triphosphate ($PIP_3$). $PIP_3$ initiates Btk activation by targeting the kinase to the plasma membrane through interactions with the Btk PH domain, a pathway which is inhibited by the activity of the *Src* homology 2 containing inositol polyphospatase SHIP (Bolland et al., 1998; Scharenberg et al., 1998; Pearse et al., 1999). In concert with this phosphatidylinositol (PI) 3-kinase and $PIP_3$-dependency, Btk activity is regulated by the α-subunit of the $G_q$ class of G proteins, and the *Src* family kinases (Bence et al., 1997, Li et al., 1997, Rawlings et al., 1996). Upon BCR or IL-5R stimulation in B cells and FcεRI in mast cells, *Src* family kinases rapidly induce phosphorylation of Y551 in the Btk kinase domain. This phosphorylation is followed by an autophosphorylation at Y223 in the SH3 domain (Wahl et al., 1997). These concerted phosphorylation events were shown to be enhanced by an E41K mutation (Glu-to-Lys) in the PH domain of Btk (Park et al., 1996). The E41K Btk mutant, isolated using a retroviral random mutagenisis scheme, was able to induce transformation of NIH 3T3 fibroblast in soft agar cultures and relieved the IL-5 dependence of pro-B cell line Y16 (Li et al., 1995). The nature of E41K transforming activity is associated with an increased membrane localization (Li et al., 1995; Varnai, et al., 1999), thereby positioning Btk in close proximity to other signaling molecules, needed for activation.

To identify B cell signaling pathways activated by Btk *in vivo* we generated transgenic mice, which express an E41K human Btk mutant. When expression was driven by the CD19 promoter, B cell development was arrested within the immature $IgM^+IgD^-$ B cell stage in the bone marrow, irrespective of the presence or absence of the endogenous intact murine *Btk* gene (Maas et al., 1999). The arrest occurred at the progression from $IgM^{low}$ into $IgM^{high}$ B cells, which reflects the first immune tolerance checkpoint at which autoreactive B cells become susceptible to apoptosis. Whereas the numbers of peripheral mature B cells in spleen and lymph nodes were reduced to < 1% of the normal numbers, a significant population of $IgM^+$ plasma cells was present in the spleen. Serum levels of IgM were substantial and increased with age (Maas et al., 1999).

A different phenotype was observed when the E41K mutant was expressed under the control of the MHC class II Ea locus control region (Dingjan et al., 1998). These mice did not exhibit any detectable defects in developing B cells in the bone marrow, but manifested a deficiency of recirculating B cells. A marked reduction of the B cell compartment was found in the spleen. Furthermore, the mice manifested a disorganization of the B cell areas and marginal zones in the spleen. In the spleen, B cell areas typically contained unusually high numbers of T cells and the T-cell area-associated $CD11c^+$ interdigitating dendritic cells, which normally do not extend into B cell follicles. These findings suggested that the expression of the E41K mutant in peripheral B cells results in follicular exclusion, followed by apoptosis for the majority of peripheral B cells in the spleen. In the lymph nodes, peripheral blood and peritoneal cavity only very few B cells were present. Furthermore, the expression of the E41K mutant was shown to enhance blast formation of purified splenic B cells *in vitro* in response to anti-IgM or LPS stimulation.

The differences between the two $Btk^{E41K}$ expressing mouse strains most likely reflect the earlier expression during B cell development of the transgene driven by the CD19 promoter region. The MCHII-h$Btk^{E41K}$ expressing cells may well escape negative selection in the bone marrow because the expression level of the transgene had not reached a critical threshold value. Due to the nature of the MHC class II Ea LCR, transgenic $Btk^{E41K}$ expression is significantly upregulated only after cells have arrived in the spleen (Dingjan et al., 1998; Maas et al., 1999).

### ROLE FOR BTK IN THE INDIVIDUAL STEPS OF B CELL DEVELOPMENT

### Btk is not required in pro and large pre-B cells

Several lines of evidence indicate that Btk is not critical for the assembly of the μ H chain and the transition from the pro-B to the small pre-B cell stage. (1) Pre-B cells are generally present at normal numbers in XLA patients (Sideras and Smith, 1995). (2) In heterozygous $Btk^{+/-}$ female mice, the absence of Btk did not result in a selective disadvantage up to the small pre-B cell stage (Hendriks et al., 1996). (3) The *xid* phenotype can be corrected by transgenic expression of Btk from the pre-B cell stage onwards (Drabek et al., 1997). (4) Pre-B cell receptor-mediated events, such as allelic exclusion and proliferation of B cell precursors that have performed a successful Ig H chain rearrangement, proceed normally in *xid* or XLA (Sideras and Smith, 1995). (5) Despite high levels of E41K mutated Btk in the pro-B and pre-B stages, the CD19-*hBtk*$^{E41K}$ transgenic mice showed defects only from the immature B cell stage onwards (Maas et al., 1999). Also when these mice were crossed on a RAG-1-deficient background, activated Btk did not signal developmental progression of pro-B lymphocytes (R.W.H., unpublished results).

### Btk is critical for the transition from the pre-B to the immature-B cell stage

The first role for Btk is evident at the transition of the small resting pre-B cell stage to the $IgM^{low}IgD^-$ immature B cell stage (Figure 2). This transition is affected both in XLA patients (Conley, 1985) and in mice, in which B cells that lack Btk exhibited a selective disadvantage compared to B cells that express Btk (Hendriks et al., 1996). These findings suggest that Btk may be an essential transducer of signals that govern Ig L chain rearrangement events, such as chromatin structure changes that allow the recombinase access to the Ig L chain gene segments. Alternatively, Btk-mediated signals may regulate the re-expression of the *RAG* gene products, which are absent in the large cycling pre-B cells and reactivated in small resting pre-B cells for Ig L chain rearrangement (Grawunder et al., 1995). Finally, Btk signaling may regulate the survival of immature B cells that have performed a successful Ig L chain rearrangement. This would be supported by the finding that Bcl-2 expression is reduced and surface Ig mediated $Bcl\text{-}x_L$ induction is absent in xid B cells (Anderson et al., 1996; Woodland et al., 1996; Solvason et al., 1998).

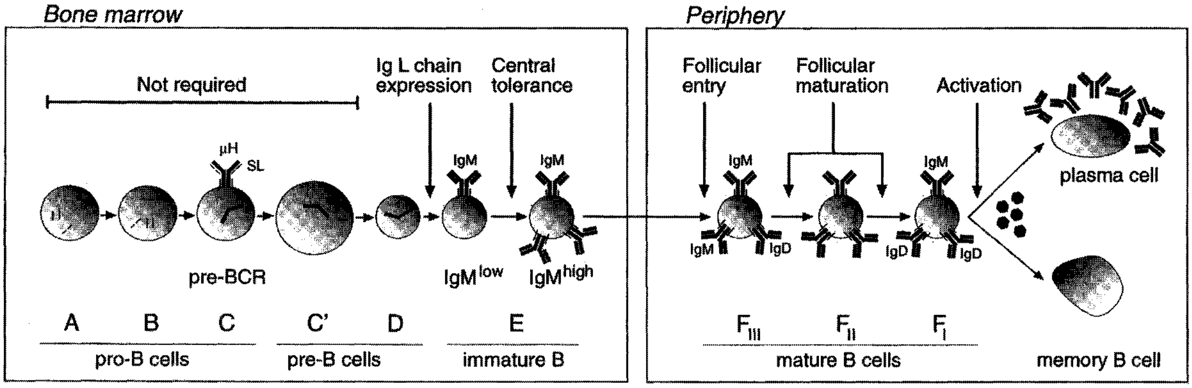

FIGURE 2 Role of Btk in murine B cell development. The model of B cell development is based on the nomenclature according to Hardy et al., 1995. The stages at which there is evidence for a role of Btk are indicated

### A role for Btk in central immune tolerance

With the expression of a complete IgM molecule and the resultant antigen specificity on the cell surface, immature B cells become susceptible to immune tolerance (Goodnow, 1996). Recently, two subpopulations within the immature B cell stage with differences in apoptosis sensitivity were described (Melamed et al., 1998). Upon BCR stimulation *in vitro*, $IgM^{low}IgD^{-}$ immature B cells performed secondary L chain rearrangements, a process termed receptor editing. Under these conditions, the slightly more mature $IgM^{high}IgD^{-}$ B cells were susceptible to apoptosis. The expression of the activated $Btk^{E41K}$ mutant driven by the CD19 promoter resulted in an almost complete absence of $IgM^{high}IgD^{-}$ immature B cells, while $IgM^{low}$IgD- cells were still present (Maas et al., 1999). Although the possibility that constitutive activation of Btk by the E41K mutation leads to a general defect that impedes the survival or affects their developmental capacities cannot be excluded, it is attractive to hypothesize that expression of $Btk^{E41K}$ mimics B cell receptor engagement. The findings in the CD19-*hBtk*$^{E41K}$ mouse would then imply that Btk is involved in the BCR signal pathway that eliminates auto-reactive B cells at the $IgM^{high}IgD^{-}$ B cell stage in the bone marrow.

### Btk-mediated signals guide follicular entry, maturation and survival

Mice in which E41K Btk expression is driven by the MHC class II LCR display normal B cell development in the bone marrow, but manifest a deficiency of recirculating follicular B cells (Dingjan et al., 1998). The B cells that were present in the spleen had an $IgM^{high}HSA^{high}B220^{low}$ surface phenotype, resembling immature cells that have recently left the bone marrow. In this respect, these cells paralleled B cells that are autoreactive for antigens present in the periphery, which are excluded from follicles and eliminated (Russel et al., 1991, Eibel et al., 1994, Goodnow et al., 1995). It is likely that the activated state of the MHCII-h*Btk*$^{E41K}$ B cells may result in an inhibition of follicular entry. As T helper cell-derived rescue signals are absent, follicular exclusion will result in apoptosis of most of the B cells. In summary, the findings in the MHCII-h*Btk*$^{E41K}$ mice imply that Btk-mediated BCR signals are decisive for the choice between follicular entry and follicular exclusion. This would be consistent with the block of B cell follicular entry in mice with targeted mutations in other BCR signaling components, such as Ig-α or Syk, (Torres et al., 1996, Turner et al., 1995).

After follicular entry, B cells are positively selected to become long-lived $IgM^{low}IgD^{high}$ recirculating B cells, a process which is probably mediated by low-level BCR signaling (Gu et al., 1991, Lam et al., 1997). Btk-deficient B cells are not excluded from B cell follicles, but fail to develop from the immature $IgM^{high}IgD^{low}$ stage into the long-lived recirculating follicular $IgM^{low}IgD^{high}$ B cell stage (Wicker and Scher, 1986, Khan et al., 1995, Hendriks et al., 1996). As *xid* B cells were shown to express only low levels of the anti-apoptotic bcl-2 protein and to undergo spontaneous apoptosis more rapidly than wild-type B cells *in vitro* (Woodland et al., 1996), we conclude that Btk must play a critical role in survival and maturation into long-lived recirculating B cells.

### Btk and plasma cell differentiation

Another role for Btk was apparent from in the CD19-h$Btk^{E41K}$ transgenic mice. As the block at the $IgM^{high}IgD^{-}$ immature B cell stage was leaky, B cells were found in the peripheral organs in very low numbers, almost exclusively bearing an $IgM^{high}HSA^{high}B220^{low}$ immature phenotype. Despite the very severe reduction of the mature B cell pool, significant numbers of IgM secreting plasma cells were present in the splenic red pulp. Therefore, we conclude that Btk activation quite efficiently induced terminal differentiation of the residual B cells into IgM-producing plasma cells, apparently without functional selection. In the CD19-h$Btk^{E41K}$ transgenic mice, serum levels of the IgG and IgA subclasses were severely decreased, confirming that constitutive BCR signaling in the absence of co-stimulation by CD40-CD40L interactions did not induce B cells to perform IgH chain class switch or germinal center formation (Foy et al., 1996).

### CONCLUDING REMARKS

The analyses of the various mouse models generated show that Btk is expressed throughout B cell development and that signaling cascades activated by Btk are critical at several checkpoints throughout B cell differentiation. In all maturation steps the strength of the BCR-mediated signal is critical to guide further development. In the absence of Btk and – even more so – in the case of constitutive activation, B cell development is impaired. The molecular mechanisms, by which Btk mediates B cell development, cell activation and cell death need to be further elucidated, and await a detailed characterization of downstream signaling targets and pathways. Very likely, the proteins that interact with the different domains of Btk vary between the individual stages of B cell development. The biochemical characterization of downstream signaling targets at these individual stages could be facilitated by the generation of transgenic mice that express tagged forms of the Btk protein. Similar approaches could be used for other components of the BCR signaling pathways and would eventually lead to a better insight in B cell development and function.

***Acknowledgements***

These studies were partially supported by grant NWO 901–07–224 from the Netherlands Organization for Scientific Research (to A.M), as well as by the Royal Academy of Arts and Sciences (to R.W.H).

***References***

Anderson, J.S., Teutsch, M., Dong, Z. and Wortis, H.H. (1996) An essential role for Bruton's tyrosine kinase in the regulation of B-cell apoptosis. *Proc. Natl. Acad. Sci. USA* **93**:10966–10971.

Aoki, Y., Isselbacher, K.J. and Pillai, S. (1994). Bruton tyrosine kinase is tyrosine phosphorylated and activated in pre-B lymphocytes and receptor-ligated B cells. *Proc. Natl. Acad. Sci. USA* **91**:10606–10609.

Bence, K., Ma, W., Kozasa, T. and Huang, X.Y. (1997). Direct stimulation of Bruton's tyrosine kinase by $G_q$-protein α-subunit. *Nature* **389**:296–299.

Bolland, S., Pearse, R.N., Kurosaki, T. and Ravetch, J.V. (1998) SHIP modulates immune receptor responses by regulating membrane association of Btk. *Immunity* **8**:509–16.

Bona, C., Mond, J.J. and Paul, W.E. (1980). Synergistic genetic defect in B-lymphocyte funtion. I. Defective responses to B-cell stimulants and their genetic basis. *J. Exp. Med.* **151**:224–234.

Bykowsky, M.J., Haire, R.N., Ohta, Y., Tang, H., Sing, S.S., Veksler, E.S., Green, J.M., Fu, S.M., Litman, G.W. and Sullivan, K.E. (1996). Discordant phenotype in siblings with X-linked agammaglobulinemia. *Am. J. Hum. Genet.* **58**:477–483.

Campana, D., Farrant, J., Inamidar, N., Webster, D.B. and Janossy, G. (1990). Phenotypic features and proliferative activity of B

cell progenitors in X-linked agammaglobulinemia. *J. Immunol.* **145**:1675–1680.

Conley, M.E., (1985). B cells in patients with X-linked agammaglobulinemia. *J. Immunol.* **134**:3070–3074.

Conley, M.E. and Cooper, M.D. (1998) Genetic basis of abnormal B cell development. *Curr. Opin. Immunol.* **10**:399–406.

Desiderio, S. (1997). Role of Btk in B cell development and signaling. *Curr. Opin. Immunol.* **9**:534–540.

De Weers. M., Verschuren, M.C.M., Kraakman, M.E.M., Mensink, R.G.J., Schuurman, R.K.B., Van Dongen, J.J.M. and Hendriks, R.W. (1993). The Bruton's tyrosine kinase gene is expressed throughout B cell differentiation from early precursor B cell stages preceding immunoglobulin gene rearrangement up to mature B cell stages. *Eur. J. Immunol.* **23**:3109–3114.

De Weers, M., Mensink, R.G., Kraakman, M.E., Schuurman, R.K.B. and Hendriks, R.W. (1994a) Mutation analysis of the Bruton's tyrosine kinase gene in X-linked agammaglobulinemia: identification of a mutation which affects the same codon as is altered in immunodeficient xid mice. *Hum. Mol. Genet.* **3**:161–166.

De Weers, M., Brouns, G.S., Hinshelwood, S., Kinnon, C., Schuurman, R.K.B., Hendriks, R.W. and Borst, J. (1994b) B-cell antigen receptor stimulation activates the human Bruton's tyrosine kinase, which is deficient in X-linked agammaglobulinemia. *J. Biol. Chem.* **269**:23857–23860.

Dingjan, G.M., Maas, A., Nawijn, M.C., Smit, L., Voerman, J.S.A., Grosveld, F. and Hendriks, R.W. (1998). Severe B cell deficiency and disrupted splenic architecture in transgenic mice expressing the E41K mutated form of Bruton's tyrosine kinase. *EMBO J.* **17**:5309–5320.

Drabek, D., Raguz, S., De Wit, T.P.M., Dingjan, G.M., Savelkoul, H.F.J., Grosveld, F. and Hendriks, R.W. (1997) Correction of the X-linked immunodeficiency phenotype by transgenic expression of human Bruton's tyrosine kinase under the control of the class II major histocompatibility complex Ea locus control region. *Proc. Natl. Acad. Sci. USA* **94**:610–615.

Eibel, H., Fiedler, P. and Köhler, G. (1994). transgenic mouse model for peripheral B cell tolerance. In "Transgenesis and targeted mutagenisis in Immunology", H. Bluethmann and P. Ohashi, Ed. (Academic Press, San Diego), pp. 251–265.

Fluckinger, A.C., Li, Z., Kato, R.M., Wahl, M.I., Ochs, H.D., Longnecker, R., Linet, J.P., Witte, O.N., Scharenberg, A.M. and Rawlings, D.J. (1998). Btk/Tec kinases regulate sustained increases in intracellular $Ca^{2+}$ following B-cell receptor activation. *EMBO J.* **17**:1973–1985.

Foy, T.M., Aruffo, A., Bajorath, J., Buhlmann, J.E. and Noelle, R.J. (1996). Immune regulation by CD40 and its ligand GP39. *Annu. Rev. Immunol.* **14**:591–617.

Genevier, H.C., Hinshelwood, S., Caspar, H.B., Rigley, K.P., Brown, D., Sealand, S., Rousset, F., Levinsky, R.J., Callard, R.E., Kinnon, C. and Lovering, R.C. (1994). Expression of Bruton's tyrosine kinase protein within the B cell lineage. *Eur. J. Immunol.* **24**:3100–3105.

Goodnow, C.C. (1996). Balancing immunity and tolerance: deleting and tuning lymphocyte repertoires. *Proc. Natl. Acad. Sci. USA* **93**:2264–2271.

Goodnow, C.C., Cyster, J.G., Hartley, S.B., Bell, S.E., Cooke, M.P., Healy, J.I., Akkarju, S., Ratmell, J.C., Pogue, S.L. and Shokat, K.P. (1995). Self-tolerance checkpoints in B lymphocyte development, *Adv. Immunol.* **59**:279–368.

Grawunder, U., Leu, T.M.J., Schatz, D.G., Werner, A., Rolink, A.G., Melchers, F. and Winkler, T.H. (1995). Down-regulation of RAG1 and RAG2 gene expression in pre-B cells after functional immunoglobulin heavy chain rearrangement. *Immunity* **3**:601–608.

Gu, H., Tarlinton, D., Müller, W., Rajewski, K. and Förster, I. (1991). Most peripheral B cells in mice are ligand-selected. *J. Exp. Med.* **173**:1357–1371.

Hardy, R.R. and Hayakawa, K. (1995) B-lineage differentiation stages resolved by multiparameter flow cytometry. *Ann. N. Y. Acad. Sci.* **764**:19–24.

Hendriks, R.W., de Bruijn, M.F.T.R., Maas, A., Dingjan, G.M., Karis, A. and Grosveld, F. (1996). Inactivation of Btk by insertion of LacZ reveals defects in B cell development only past the pre-B cell stage. *EMBO J.* **15**:4862–4872.

Hinshelwood, S., Lovering, R.C., Genevier, H.C., Levinsky, R.J. and Kinnon, C. (1995). The protein defective in X-linked agammaglobulinemia, Bruton's tyrosine kinase, shows increased autophosphorylation in vitro when isolated from cells in which the B cell receptor has been cross-linked. *Eur. J. Immunol.* **25**:1113–1116.

Holsinki-Feder, E., Weiss, M., Brandau, O., Jedele, K.B., Nore, B., Bäckesjö, M., Vihinen, M., Hubbard, S.R., Belohradsky, B.H., Smith, C.I.E. and Meindl, A. (1998.) Mutation screening of the Btk gene in 56 families with X-linked agammaglobulinemia (XLA): 47 unique mutations without correlation to clinical course. *Pediatrics* **101**:276–284.

Kerner, J.D., Appleby, M.W., Mohr, R.N., Chien, S., Rawlings, D.J., Maliszewski, C.R., Witte, O.N. and Perlmutter, R. (1995). Impaired expansion of mouse B cell progenitors lacking Btk. *Immunity* **3**:301–312.

Khan, W.N., Alt, F.W., Gerstein, R.M., Malynn, B.A., Larsson, I., Rathburn, G., Davidson, L., Müller, S., Kantor, A.B., Herzenberg, L.A., Rosen, F.A. and Sideras, P. (1995). Defective B cell development and function in Btk-deficient mice. *Immunity* **3**:283–299.

Lam, K. P., Kühn, R. and Rajewsky, K. (1997). In vivo ablation of surface immunoglobulin on mature B cells by inducible gene targeting results in rapid cell death. *Cell* **90**:1073–1083.

Li., T., Tsukada, S., Satterthwaite, A., Havlik, M.H., Park, H., Takatsu, K. and Witte, O.N. (1995) Activation of Bruton's tyrosine kinase (BTK) by a point mutation in its pleckstrin homology (PH) domain. *Immunity* **2**:451–460.

Li, Z.M., Wahl, M.I., Eguinoa, A., Stephens, L.R., Hawkins, P.T. and Witte, O.N. (1997). Phosphatidylinositol 3-kinase-gamma activates Bruton's tyrosine kinase in concert with Src family kinases. *Proc. Natl. Acad. Sci. USA* **94**:13820–13825.

Maas, A., Dingjan, G.M., Grosveld, F. and Hendriks, R.W. (1999) Early arrest in B cell development in transgenic mice that express the E41K Bruton's tyrosine kinase mutant under the control of the CD19 promoter region. *J. Immunol.* 162:6526–6533.

Maas, A., Dingjan, G.M., Savelkoul, H.F.J., Kinnon, C., Grosveld, F. and Hendriks, R.W. (1997). The X-linked immunodeficiency defect in the mouse is corrected by expression of human Bruton's tyrosine kinase from a yeast artificial chromosome transgene. *Eur. J. Immunol.* **27**:2180–2187.

Melamed, D., Benschop, R.J., Cambier, J.C., and Nemazee, D. (1998). Developmental regulation of B lymphocyte immune tolerance compartmentalizes clonal selection from receptor editing. *Cell* **92**:173–182.

Mohamed, A.J., Nore, B.F., Christensson, B. and Smith, C.I. (1999) Signalling of Bruton's tyrosine kinase, Btk. *Scand. J. Immunol.* **49**:113–118.

Mukhopadhyay, S., George, A., Bal, V., Ravindran, B. and Rath, S. (1999) Bruton's tyrosine kinase deficiency in macrophages inhibits nitric oxide generation leading to enhancement of IL-12 induction. *J. Immunol.* **163**:1786–1792.

Nonoyama, S., Tsukada, S., Yamadori, T., Miyawaki, T., Jin, Y.Z., Watanabe, C., Morio, T., Yata, J. and Ochs, H.D. (1998). Functional analysis of peripheral blood B cells in patients with X-linked agammaglobulinemia. *J. Immunol.* **161**:3925–3929.

Oeltjen, J.C., Malley, T.M., Muzny, M., Miller, W., Gibbs, R.A. and Belmont, J.W. (1997). Large-scale comparative sequence analysis of the human and murine Bruton's tyrosine kinase loci reveals conserved regulatory domains. *Genome Res.* **7**:315–329.

Park, H., Wahl, M.I., Afar, D.E.H., Turck, C.W., Rawlings, D.J., Tam, C., Scharenberg, A.M., Kinet, J.-P. and Witte, O.N. (1996). Regulation of Btk function by a major autophosphorylation site within the SH3 domain. *Immunity* **4**:515–525.

Pearse, R.N., Kawabe, T., Bolland, S., Guinamard, R., Kurosaki, T. and Ravetch, J.V. (1999) SHIP recruitment attenuates Fc gamma RIIB-induced B cell apoptosis. *Immunity* **10**:753–60.

Quek, L.S., Bolen, J., Watson, S.P. (1998) A role for Bruton's tyrosine kinase (Btk) in platelet activation by collagen. *Curr Biol* **8**:1137–1140.

Rawlings, D.J., Saffran, D.C., Tsukada, S., Largaespada, D.A., Grimaldi, J.C., Cohen, L., Mohr, R.N., Bazan, J.F., Howard, M., Copeland, M.G., Jenkins, N.A. and Witte, O.N. (1993). Mutation of the unique region of Bruton's tyrosine kinase in immunodeficient mice. *Science* **261**:358–361.

Rawlings, D.J. Scharenberg, A.M., Park, H., Wahl, M.I., Lin, S., Kato, R.M., Fluckinger, A.-C., Witte, O.N. and Kinet, J.-P. (1996). Activation of BTK by a phosphorylation mechanism initiated by SRC family kinases. *Science* **271**:822–825.

Russell, D.M., Dembic, Z., Morahan, G., Miller, J.F.A.P., Bürki, K. and Nemazee, D. (1991). Peripheral deletion of self-reactive B cells. *Nature* **354**:308–311.

Saouf, S.J., Mahajan, s., Rowley, R.B., Kut, S.A., Fargnoli, J., Burkhardt, A.L., Tsukadà, S., Witte, O.N. and Bolen, J.B. (1994). Temporal differences in the activation of three classes of non-transmembrane protein tyrosine kinases following B-cell antigen receptor surface engagement. *Proc. Natl. Acad. Sci. USA* **91**:9524–9528.

Satterthwaite, A. and Witte, O. (1996). Genetic analysis of tyrosine kinase function in B cell development. *Annu. Rev. Immunol.* **14**:131–154.

Satterthwaite, A.B., Cheroutre, H., Khan, W.N., Sideras, P. and Witte, O.N. (1997). Btk dosage determines sensitivity to B cell antigen receptor cross-linking. *Proc. Natl. Acad. Sci. USA* **94**:13152–13157.

Scharenberg, A.M., El-Hillal, O., Fruman, D.A., Beitz, L.O., Li, Z., Lin, S., Gout, I., Cantley, L.C., Rawlings, D.J. and Kinet, J.P. (1998) Phosphatidylinositol-3,4,5-trisphosphate (PtdIns-3,4,5-P3)/Tec kinase-dependent calcium signaling pathway: a target for SHIP-mediated inhibitory signals. *EMBO J.* **17**:1961–1972.

Sideras, P. and Smith, C.I.E. (1995). Molecular and cellular aspects of X-linked agammaglobulinemia. *Adv. Immunol.* **59**:135–223.

Smith, C.I.E., Baskin, B., Humire-Greiff, P., Zhou, J-N., Olsson, P.G., Maniar, H.S., Kjellén, P., Lambris, J.D., Christensson, B., Hammarström, L., Bentley, D., Vetrie, D., Islam, K.B., Vorechovsky, I. and Sideras, P. (1994). Expression of Bruton's agammaglobulinemia tyrosine kinase gene, Btk, is selectively down-regulated in T lymphocytes and plasma cells. *J. Immunol.* **152**:557–565.

Solvason, N., Wu, W.W., Kabra, N., Lund-Johansen, F., Roncarolo, M.G., Behrens, T.W., Grillot, D.A., Nunez, G., Lees, E. and Howard, M. (1998) Transgene expression of bcl-xL permits anti-immunoglobulin (Ig)-induced proliferation in xid B cells. *J. Exp. Med.* **187**:1081–1091.

Takata, M. and Kurosaki, T. (1996). A role for Bruton's tyrosine kinase in B cell antigen receptor-mediated activation of phospholipase C-γ2. *J. Exp. Med.* **184**:31–40.

Thomas, J.D., Sideras, P., Smith, C.I.E., Vorechovsky, I., Chapman, V. and Paul, W.E. (1993). Colocalization of X-linked agammaglobulinemia and X-linked immunodefiecieny genes. *Science* **261**:355–358.

Torres, R.M., Flaswinkel, H., Reth, M. and Rajewski, K. (1996). Aberant B cell development and immune responses in mice with a compromised BCR complex. *Science* **272**:1804–1808.

Tsukada, S., Saffran, D.C., Rawlings, D.J., Parolini, O., Allen, R.C., Klisak, I., Sparkes, R.S., Kubagawa, H., Thuluvancheri, M., Quan, S., Belmont, J.W., Cooper, M.D., Conley, M.E. and Witte, O.N. (1993). Defecient expression of a B cell cytoplasmic tyrosine kinase in human X-linked agammaglobulinemia. *Cell* **72**:279–290.

Turner, M., Mee, P.J., Costello, P.S., Williams, O., Price, A.A., Duddy, L.P., Furlong, M.T., Geahlen, R.L. and Tybulewicz, V.L. (1995). Perinatal lethality and blocked B-cell development in mice lacking the tyrosine kinase Syk. *Nature* **378**:298–302.

Varnai, P., Rother, K.I. and Balla, T. (1999) Phosphatidylinositol 3-kinase-dependent membrane association of the Bruton's tyrosine kinase pleckstrin homology domain visualized in single living cells. *J. Biol. Chem.* **274**:10983–10989.

Vetrie, D., Vorechovsky, I., Sideras, P., Holland, J., Davies, A., Flinter, F., Hammarström, L., Kinnon, C., Levinsky, R., Bobrow, M., Smith, C.I.E. and Bentley, D.R. (1993). The gene involved in X-linked agammaglobulinemia is a member of the src family of protein kinases. *Nature* **361**:226–223.

Vihinen, M., Kwan, S.P., Lester, T., Ochs, H.D., Resnick, I., Valiaho, J., Conley, M.E., Smith, C.I. (1999) Mutations of the human BTK gene coding for bruton tyrosine kinase in X-linked agammaglobulinemia. *Hum. Mutat.* **13**:280–5.

Wahl, M.I., Fluckinger, A-C., Kato, R.M., Park, H., Witte, O.N. and Rawlings, D.J. (1997). Phosphorylation of two regulatory tyrosine residues in the activation of Bruton's tyrosine kinase via alternative receptors. *Proc. Natl. Acad Sci. USA* **94**:11526–11533.

Wicker, L.S. and Scher, I. (1986). X-linked immune deficiency (xid) of CBA/N mice. *Curr. Top. Microbiol. Immunol.* **124**:87–101.

Woodland, R.T., Schmidt, M.R., Korsmeyer, S.J. and Gravel, K.A. (1996). Regulation of B cell survival in xid mice by the proto-oncogene bcl-2. *J. Immunol.* **156**:2143–2154.

